# Narrowing the gap between machine learning scoring functions and free energy perturbation using augmented data

**DOI:** 10.1038/s42004-025-01428-y

**Published:** 2025-02-08

**Authors:** Ísak Valsson, Matthew T. Warren, Charlotte M. Deane, Aniket Magarkar, Garrett M. Morris, Philip C. Biggin

**Affiliations:** 1 Oxford Protein Informatics Group, Department of Statistics, University of Oxford, Oxford, UK; 2 Structural Bioinformatics and Computational Biochemistry, Department of Biochemistry, University of Oxford, Oxford, UK; 3 Boehringer Ingelheim Pharma GmbH & Co. KG, Biberach an de Riß, Germany

**Keywords:** Cheminformatics, Virtual screening, Lead optimization, Method development

## Abstract

Machine learning offers great promise for fast and accurate binding affinity predictions. However, current models lack robust evaluation and fail on tasks encountered in (hit-to-) lead optimisation, such as ranking the binding affinity of a congeneric series of ligands, thereby limiting their application in drug discovery. Here, we address these issues by first introducing a novel attention-based graph neural network model called AEV-PLIG (atomic environment vector–protein ligand interaction graph). Second, we introduce a new and more realistic out-of-distribution test set called the OOD Test. We benchmark our model on this set, CASF-2016, and a test set used for free energy perturbation (FEP) calculations, that not only highlights the competitive performance of AEV-PLIG, but provides a realistic assessment of machine learning models with rigorous physics-based approaches. Moreover, we demonstrate how leveraging augmented data (generated using template-based modelling or molecular docking) can significantly improve binding affinity prediction correlation and ranking on the FEP benchmark (weighted mean PCC and Kendall’s *τ* increases from 0.41 and 0.26 to 0.59 and 0.42). These strategies together are closing the performance gap with FEP calculations (FEP+ achieves weighted mean PCC and Kendall’s *τ* of 0.68 and 0.49 on the FEP benchmark) while being  ~400,000 times faster.

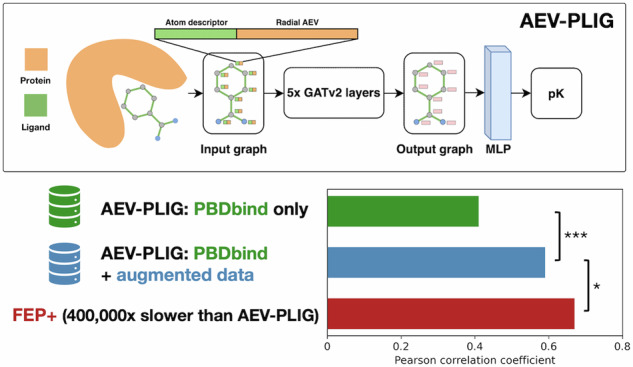

## Abstract (web summary)

Machine learning offers great promise for fast and accurate binding affinity predictions, however, current models lack robust evaluation and fail on tasks encountered in drug discovery. Here, the authors introduce an attention-based graph neural network model, AEV-PLIG, and demonstrate that augmenting the training data with semi-synthetic data significantly improves AEV-PLIG’s performance on benchmarks used for free energy perturbation calculations, narrowing the performance gap to simulation-based methods.

## Introduction

Predicting the change in free energy upon binding of a protein and a ligand represents a cornerstone of computational small molecule drug discovery. It is essential during hit identification, where one aims to identify binders that demonstrate high affinity for a target, as well as in hit-to-lead and lead optimisation, where binding affinity must be optimised alongside a number of other properties pertinent to safety and biological efficacy. Given the vastness of chemical space—and hence possible drug candidates^1^—computer-aided drug design (CADD) can play an important role in accelerating the discovery process by enabling large numbers of compounds to be screened in silico, avoiding the significant cost and resources required for experimental measurements^2^. The need for fast and accurate virtual screening methods is self-evident.

To predict binding affinity, computational methods typically employ knowledge- or physics-based approaches that depend on statistical potentials or those based on molecular mechanics force fields, respectively^3^. These approaches offer a trade-off between computational cost and accuracy: fast estimations of binding affinity, such as scoring functions used in molecular docking, rely on heuristics and physical approximations that can limit their accuracy^4^. In contrast, alchemical binding free energy (BFE) simulation methods using all-atom molecular dynamics (MD) in explicit solvent offer a more rigorous but more expensive approach to compute either the absolute binding free energy (ABFE) of a ligand and protein^5^ or the relative binding free energy (RBFE) between two similar ligands that bind to the same protein^6^. A popular class of alchemical method, namely free energy perturbation (FEP)^7^ theory, often performed using the FEP+ workflow^8^, has demonstrated performance approaching the limits of achievable chemical accuracy (~1 kcal/mol) for certain systems^9,10^. However, FEP (herein used interchangeably with alchemical BFE calculations) suffers from several limitations, including: a strong dependence on the choice of MD force field^9,11^; a need for extensive and custom preparation (although see ref. ^12^ and ref. ^13^ for recent progress in this area) and parameterisation protocols^6^; and restrictions on the nature and extent of structural modifications that are permitted between two ligands in RBFE calculations. Moreover, the computational cost of FEP limits molecular throughput and is thus prohibitive for high-throughput virtual screening.

Machine learning (ML) has emerged as a promising alternative for fast and accurate binding affinity prediction, benefitting from ever-increasing quantities of both binding affinity measurements and high-resolution structural data^14^. By training models using large datasets of protein–ligand complex structures labelled with their corresponding experimental affinity measurements, ML algorithms are trained to learn a mapping between molecular structure or features of the complex and binding affinity. Once trained, these models can then predict the binding affinity of new protein–ligand complexes from their structure alone using a fraction of the time and compute required for simulation-based methods. The past decade has seen rapid growth in the development of ML scoring functions using a variety of protein–ligand representations, such as intermolecular interaction fingerprints^15^, extended connectivity fingerprints^16^, and ligand-based physicochemical descriptors^17,18^. More recently, deep learning architectures that do not rely on explicit featurisation, such as convolutional neural networks (CNNs)^19^ and graph neural networks (GNNs)^20,21^, have emerged. Typically, ML models are formulated as regression tasks trained on the PDBbind dataset^22^, which in version 2020 contains around 20,000 curated protein–ligand complex structures with associated binding affinity measurements. To evaluate their performance, the models are then tested with complexes from the critical assessment of scoring function (CASF) benchmarks^23,24^. In these scenarios, deep learning models have been shown to achieve strong performance compared to traditional scoring functions (PCC range of 0.85–0.90, and RMSE range of 1.5–2.0 kcal/mol)^14,19,20^.

Given this impressive performance, we might expect that such models have learnt the physical principles of protein–ligand interactions such that they can generalise to protein–ligand complexes not seen during training, and ultimately predict the result of subtle chemical changes in binding affinity. However, a number of analyses have shown that current models frequently fail to learn key biophysical principles, evidenced by their poorer performance on out-of-distribution (OOD) datasets, implying memorisation of ligand-based features^25–27^ or even fitting to noise^28^. This issue is compounded by the fact that deep learning models are typically “black box” systems, making it challenging to interpret their predictions and interrogate the knowledge learnt during training. Consequently, the application of ML scoring functions beyond benchmarks in “real world” drug discovery pipelines has been limited, and more expensive methods such as FEP remain the gold standard for accurate and reliable binding affinity predictions. One of the first steps in remedying this problem is to construct and assess methods using benchmarks that are OOD.

However, while the development of novel ML algorithms and architectures has led to some improvement in prediction accuracy, significant progress has been stifled by a fundamental lack of data. Structure-based methods require both accurate binding affinity measurements and the corresponding three-dimensional (3D) structures of protein–ligand complexes (especially the binding pocket) solved at sufficient resolution. One strategy to increase the amount and diversity of training data is to use data augmentation, wherein synthetic data is generated to mimic real-world observations or by modifying existing experimentally determined examples. This approach has proven effective for applications in computer vision^29^ and natural language processing^30^, however, generating meaningful biological and chemical data de novo can be challenging due to its inherent complexity and physicochemical constraints.

In this work, we propose strategies that improve the applicability of ML scoring functions while providing more realistic assessments of their performance. First, we introduce a novel featurisation of protein–ligand interactions that combine atomic environment vectors (AEVs)^31,32^ with protein–ligand interaction graphs (PLIGs). The resulting method, termed AEV-PLIG, uses an expressive attentional GNN architecture that learns the relative importance of neighbouring environments in order to capture the complex and nuanced interactions between protein and ligand atoms^33^. Second, we construct a new out-of-distribution benchmark (OOD test) that is designed to penalise ligand and/or protein memorisation, unlike widely-used benchmarks such as CASF-2016. We use this in conjunction with other test sets that are representative of real-world drug discovery projects that feature pharmaceutically-relevant targets each with a congeneric series of ligands typical of hit-to-lead and lead optimisation campaigns^10^. Originally designed to benchmark and validate the performance of FEP, these test sets not only provide a more realistic indication of prediction accuracy for applications in CADD, but also enable a direct comparison between current ML- and MD-based approaches. To overcome the lack of training data, we also explore the use of augmented data, wherein models are trained on both 3D protein–ligand complexes with experimental binding affinity data and structures modelled using a template-based ligand alignment algorithm^34^ and molecular docking^35^. Altogether, we find that AEV-PLIG consistently shows comparable or better performance than a variety of other ML-based methods across a diverse range of benchmarks. Training with augmented data also offers a highly effective route to significantly improve prediction correlation and ranking for congeneric series typically encountered in drug discovery. Moreover, AEV-PLIG is orders of magnitude faster than FEP, requires minimal per-system preparation, and yields absolute binding affinities as opposed to relative free energy differences. This work highlights the potential of novel featurization that captures protein–ligand interactions, the need for more robust benchmarks, and the emerging role of augmented data in training ML-based scoring functions for rapid and accurate binding affinity prediction.

## Results and Discussion

### AEV-PLIG achieves competitive performance on better benchmarks

**Fig. 1:**
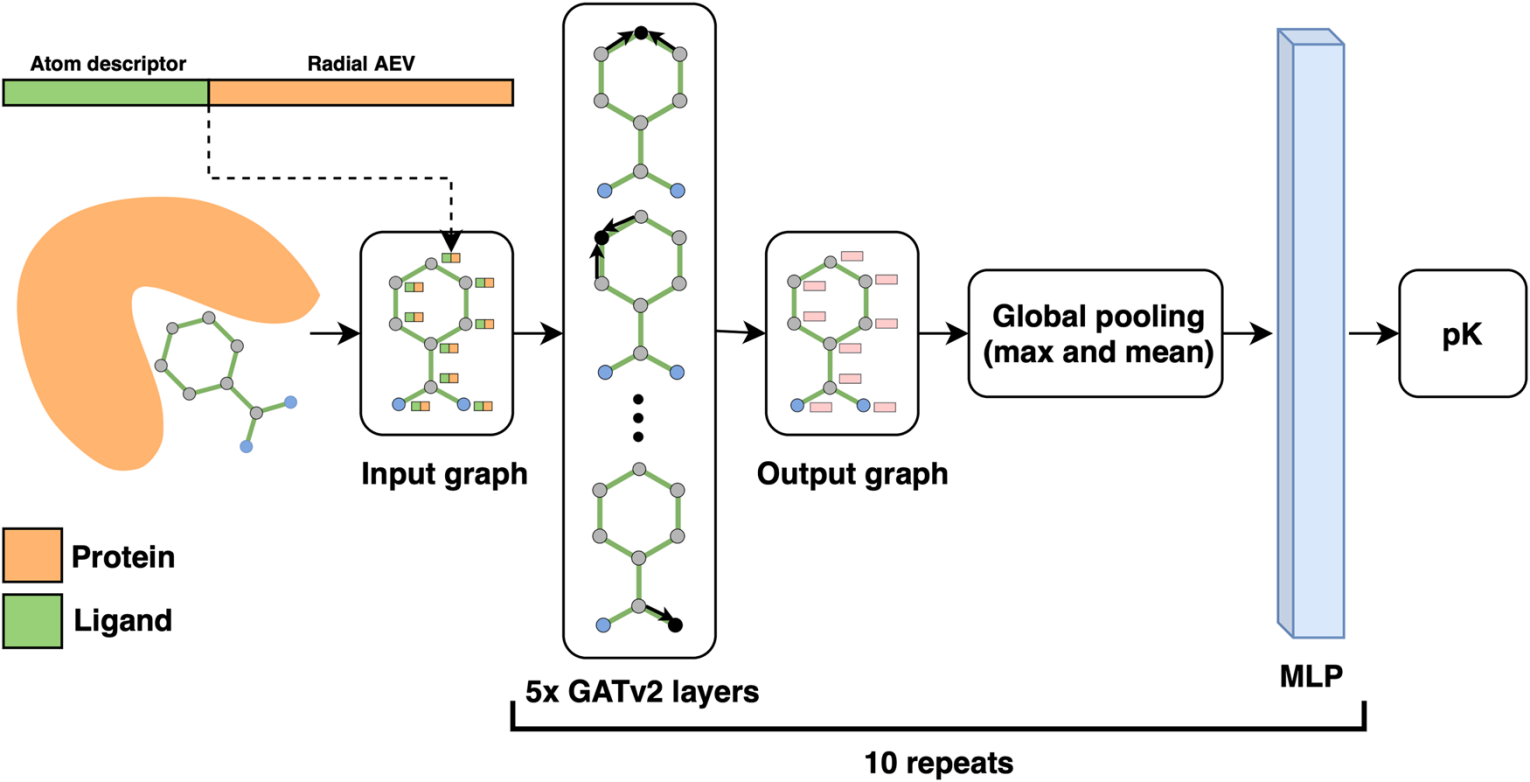
Diagram showing the AEV-PLIG architecture. The input ligand graph with node feature vectors consisting of atom descriptors and radial AEVs are propagated through five GATv2 layers^33^, each with three attention heads. Thereafter, a global pooling procedure is performed with concatenated vectors from max and mean pooling, followed by a four-layer MLP. The model prediction is calculated as the mean of the output of an ensemble of ten identical architectures that were independently trained with different seeds.

Our previous model using AEVs (AEScore^32^) showed comparable performance to other state-of-the-art ML methods, however, there was still room for improvement. This led us to the development of AEV-PLIG, a novel attention-based graph-ML scoring function illustrated in Fig. 1. Graph-based methods have emerged as a popular architecture for protein–ligand binding affinity prediction due to their ability to naturally represent molecular 3D structures and topologies^36^. Extending this representation to molecular complexes, Moesser et al. recently introduced protein–ligand interaction graphs (PLIGs) that encode intermolecular contacts between proteins and ligands as graph node features^37^. However, as the interactions in PLIGs are featured as protein atom counts determined up to a cutoff distance of the ligand atom, intermolecular distance information is not encoded. Here, AEV-PLIG builds on the concept of PLIGs by instead featuring protein–ligand interactions using ligand atom descriptors and radial atomic environment vectors (AEVs) centred on ligand atoms as the node features. AEVs are composed of atom-centred symmetry functions, which describe the local chemical environment of a reference atom using a set of Gaussian functions that depend on distances or angles between nearby atoms^31^. AEVs have been used previously for binding affinity prediction models, where they have been constructed using chemical element atom typing that does not explicitly account for atom connectivity^21,32^. Thus, we also rationalised that typing atoms using extended connectivity interaction features (ECIF)^38^, which offer a richer set of 22 distinct protein atom types, would provide a more detailed and informative representation of the chemical environment. In addition, while AEVs are typically composed of radial and angular parts describing two- and three-atom interactions respectively, AEV-PLIG uses only the radial AEVs introduced by Smith et al.^39^ that incorporate intermolecular pairwise atomic interactions. Moreover, AEV-PLIG leverages the recently developed GATv2 layers^33^, an enhanced version of GATs^40^ that makes them strictly more expressive. The graphs that feature the protein–ligand complex in AEV-PLIG are propagated through five GATv2 layers, followed by a global pooling procedure. Finally, a vector representation of the graph is passed through a four-layer multi-layer perceptron (MLP) to output a binding affinity (pK) prediction.

**Fig. 2:**
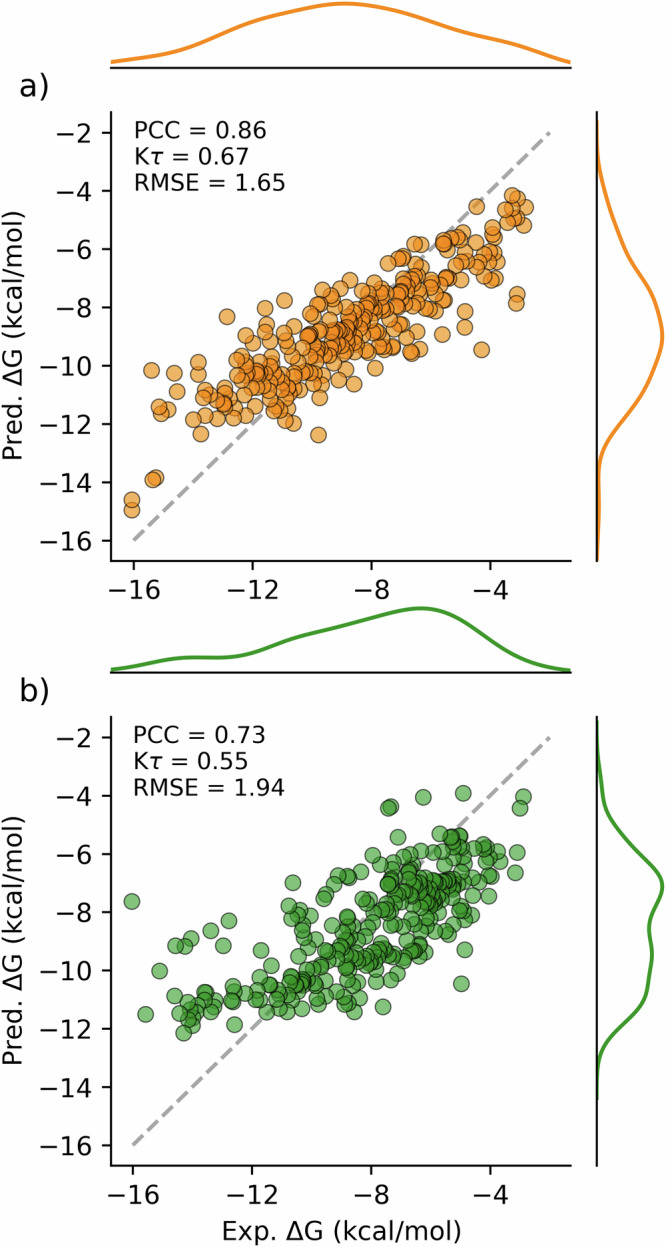
Scatter plots showing predicted vs. experimental binding free energies for AEV-PLIG models. Model trained for **a** the CASF-2016 benchmark with PDBbind v2020 and **b** for the OOD Test benchmark with Refined2020+. RMSE is given in kcal/mol.

We evaluated AEV-PLIG on the CASF dataset designed as a “scoring benchmark” to assess the predictive accuracy of computational methods^23,24^. Widely used in the development and evaluation of ML models for affinity prediction, the CASF-2016 benchmark contains 285 high-quality protein–ligand complexes representing 57 different proteins. When evaluated on this set, AEV-PLIG demonstrated excellent performance (Fig. 2, PCC = 0.86, K*τ* = 0.67), exceeding or comparable to a variety of other empirical and ML-based methods (Table 1). However, despite strong performance on this benchmark, a number of studies have highlighted that ML models frequently “memorise” training examples and overfit to biases and even noise present in this data, instead of learning the underlying biophysics^25,26,41^. These issues are exacerbated by the fact that the CASF benchmarks are contaminated, being sampled from the PDBbind dataset to ensure each protein cluster in the training data has representative test cases, thus rewarding memorisation. The differences in X-ray crystal structure resolution and ligand size (32 heavy atoms in PDBbind v2019 vs 24 in CASF-2016) may also influence the accuracy of predictions for CASF-2016 targets. These factors can lead to an over-optimistic assessment of model performance, yielding accuracy metrics that belie the model’s actual capabilities when presented with previously unseen protein–ligand complexes likely to be encountered in real-world applications. Moreover, many ML models do not explicitly encode protein–ligand interactions, which can lead to overfitting of ligand- or protein-based features present in the training set^25^.

**Table 1:** Performance metrics for AEV-PLIG and other state-of-the-art binding affinity predictors on five different benchmarks

Model	CASF-2016		OOD Test		0 Ligand Bias		FEP benchmark	
	PCC (*↑*)	K*τ* (*↑*)	PCC (*↑*)	K*τ* (*↑*)	PCC (*↑*)	K*τ* (*↑*)	wmPCC (*↑*)	wmK*τ* (*↑*)
RF-score	0.82	0.63	0.64	0.48	0.24	0.16	0.29	0.18
	[0.77, 0.85]	[0.58, 0.67]	[0.58, 0.70]	[0.42, 0.53]	[0.14, 0.34]	[0.09, 0.22]	[0.17, 0.40]	[0.10, 0.27]
Pafnucy	0.76	0.55	0.55	0.39	0.17	0.10	0.26	0.18
	[0.70, 0.80]	[0.50, 0.60]	[0.47, 0.62]	[0.32, 0.45]	[0.07, 0.28]	[0.03, 0.18]	[0.17, 0.36]	[0.12, 0.23]
OnionNet-2	0.83	0.64	0.57	0.39	0.35	**0.23**	0.27	0.18
	[0.79, 0.86]	[0.59, 0.68]	[0.49, 0.63]	[0.32, 0.45]	[0.23, 0.44]	**[0.16, 0.30]**	[0.18, 0.35]	[0.11, 0.25]
PointVS	0.80	0.60	0.69	0.51	0.28	0.17	0.33	0.22
	[0.76, 0.84]	[0.55, 0.64]	[0.63, 0.74]	[0.46, 0.56]	[0.19, 0.38]	[0.10, 0.24]	[0.23, 0.43]	[0.15, 0.29]
SIGN	0.82	0.63	0.66	0.49	0.27	0.18	0.37	**0.26**
	[0.78, 0.86]	[0.58, 0.67]	[0.59, 0.71]	[0.43, 0.54]	[0.16, 0.37]	[0.11, 0.25]	[0.29, 0.45]	**[0.21, 0.32]**
AEScore	0.85	0.64	0.63	0.44	0.26	0.17	0.28	0.21
	[0.81, 0.88]	[0.60, 0.68]	[0.57, 0.69]	[0.38, 0.49]	[0.15, 0.36]	[0.09, 0.23]	[0.19, 0.37]	[0.14, 0.28]
**AEV-PLIG**	**0.86**	**0.67**	**0.73**	**0.55**	**0.37**	0.21	**0.41**	**0.26**
	**[0.82, 0.89]**	**[0.62, 0.71]**	**[0.66, 0.77]**	**[0.50, 0.60]**	**[0.25, 0.49]**	[0.14, 0.29]	**[0.32, 0.49]**	**[0.20, 0.32]**

All models were trained using subsets of PDBbind v2020, as described in “Methods”. Performance is measured in terms of Pearson correlation coefficient (PCC) and Kendall’s *τ* (K*τ*) for CASF-2016, OOD Test, and 0 Ligand Bias. For Wang FEP and Schindler FEP, the weighted mean PCC and K*τ* over the different targets are reported, weighted by the relative number of data points per target (wmPCC and wmK*τ*, respectively). 95% Bias-corrected and accelerated (BCa) bootstrap confidence intervals are reported in square brackets and the best performing models are highlighted in bold for each metric and each test set.

To overcome this, a number of strategies to construct more representative test sets that avoid data leakage have been proposed. One solution is to use a time-based splitting procedure to separate training and test cases based on their date of publication. However, given that new drugs are often developed for existing targets and/or using existing scaffolds, this approach does not rule out overlap between training and tests. Alternatively, scaffold- or target-based splitting can be used to separate complexes based on the core molecular structure and protein sequence identity, respectively. This approach does not explicitly take into account the ligand similarity, including non-core functional groups and molecular topology. Instead, we propose a new benchmark set—the OOD test—which is constructed by minimising the similarity between the complexes in the OOD Test set and the PDBbind v2020 training data, in terms of ligand similarity, protein sequence identity, and protein pocket similarity. The resulting set contains 295 complexes from the Refined set of PDBbind v2020, with the remaining data used for training. The distributions of maximum ligand, protein sequence, and protein–ligand interaction similarities between training and test ligands are shown in Fig. S1; further details concerning this dataset and its construction are provided in “Methods”.

Binding affinity predictions obtained for the OOD Test set are shown in Fig. 2, revealing a weaker correlation (PCC) and ranking (K*τ*) than predictions for CASF-2016. This is expected, given that performance on CASF-2016 has also been shown to decrease when similar ligands and proteins are removed from the training set^18^. By providing a more realistic estimate of performance on OOD data, this benchmark also facilitates more realistic comparison and insight into the generalisation capabilities of models employing different featurization and architectures. To this end, we trained and evaluated a number of state-of-the-art models using the same training data on the OOD Test benchmark. The results, summarised in Table 1, reveal a significant drop in performance compared to CASF-2016 for all models. For OnionNet and Pafnucy, prediction correlations fall from 0.83 to 0.57, and 0.76 to 0.55, respectively, highlighting the limitations of evaluating a model on only a single benchmark.

In addition to the OOD Test, we also evaluated our models using the 0 Ligand Bias dataset devised by Durant et al.^41^ (Table 1). This benchmark consists of 365 protein–ligand complexes where the same ligand is bound to multiple different proteins, and is designed to penalise models that might learn predominantly from ligand-based features. All models tested achieved lower prediction correlation for this set compared to their performance on CASF-2016, illustrating over-fitting to ligand data. Compared to other models, AEV-PLIG achieved the best prediction correlation (PCC = 0.37); and its ranking ability (K*τ* = 0.21) was comparable to OnionNet-2, a CNN model that uses a radial encoding to capture protein–ligand interactions^42^. AEScore^32^, a feed-forward NN also trained on atomic environment vector representations, has worse performance than AEV-PLIG across all benchmarks (Table 1). However, unlike AEScore, AEV-PLIG utilises only the radial AEV components and employs ECIF atom typing rather than just elements, thus taking into account the atom connectivity explicitly when constructing atom-centred symmetry functions^38^. Combined with a graph attention network architecture, AEV-PLIG’s representation appears to enable enhanced performance.

While is it crucial to estimate OOD performance when constructing and comparing different methods, the primary application of these ML models lies in early-stage drug discovery, where we seek to identify and rank the most potent compounds among a series of related molecules with small chemical modifications designed to inhibit a specific target. It is therefore important to evaluate our models on benchmarks representative of this scenario. Thus, we also used the recent FEP benchmark set compiled by Ross and colleagues^10^. This comprehensive benchmark represents the largest publicly available collection of proteins and congeneric ligand series, covering a wide range of targets and small molecule perturbations found in drug discovery projects. In total, the dataset comprises over 50 unique protein targets and over 1200 ligands born out of hit-to-lead and lead optimisation projects^10^. In each case, RBFE calculations were performed using FEP+ and analysed retrospectively to evaluate the efficacy of this alchemical workflow in industrial applications. Additional details concerning the compilation and composition of this dataset can be found in the “Methods” and original publication, while a full list of targets is available in Table S2.

The performance of AEV-PLIG models on the FEP benchmark is shown in Table 1 as a weighted average across all series with ten or more ligands, with individual series metrics reported in Fig. S2. Compared to CASF-2016, AEV-PLIG’s binding affinity predictions for the FEP benchmark were markedly worse, with weighted mean correlation (PCC) and ranking (K*τ*) metrics of 0.41 and 0.26, respectively (Table 1). This reflects both the challenging nature of these scenarios, many of which contain structurally similar ligands with substantial differences in activity (i.e. activity cliffs) and counterintuitive structure-activity relationships. In fact, none of the ML-based or empirical methods evaluated here performed well on these systems: AEV-PLIG and SIGN yielded the most accurate predictions, but displayed only weak to moderate correlation on average (Table 1). In contrast, binding affinities computed via FEP+ had weighted average PCC and K*τ* values of 0.68 and 0.49, respectively.

Moreover, the series in the FEP benchmark typically have narrow experimental affinity ranges (Fig. S3b), making it harder to achieve high prediction correlation compared to mixed datasets that encompass many more targets and ligands, such as CASF-2016 (Fig. 2a, with a range of 14 kcal/mol or 9 pK units). For AEV-PLIG on the FEP benchmark, we found that the PCC and K*τ* values calculated for the largest series (≥25 ligands) combined were 0.56 and 0.36, respectively, compared to weighted mean values of 0.37 and 0.24 across targets individually (Fig. S3). Given that we often apply these models to score and rank similar compounds that bind to the same target of interest, series-based evaluations provide a more realistic assessment of model performance.

Altogether, the results in this section highlight the concerning discrepancy between the accuracy of binding affinity predictions for complexes in the widely used benchmark, CASF-2016, and more realistic test sets designed to probe OOD performance, penalise ligand memorisation, and validate models for lead optimization^41^. These data suggest that, in order to robustly evaluate ML models for binding affinity prediction, it is crucial to benchmark performance on diverse datasets that capture the nuances and challenges of real-world data and applications^41^. We also emphasise that while AEV-PLIG outperforms the other ML methods tested here across most benchmarks, we do not claim it is the optimal model; rather, our analysis aims to illustrate the variability in the performance of different models on different benchmarks, and highlight the strengths and weaknesses of different protein–ligand representations.

### Augmented training data improves AEV-PLIG

A major issue affecting ML model performance is the lack of diverse, high-quality training data. This is particularly challenging in biochemical applications, where obtaining new data involves costly and laborious experimental measurements, such as X-ray crystallography or isothermal titration calorimetry. Sequence-based binding affinity prediction methods have attempted to circumvent this issue using non-structure derived protein and ligand representations, enabling training on larger quantities of binding affinity data such as is accessible via ChEMBL, and is particularly valuable where the protein target sequence is known but the 3D structure is not. However, these methods have yet to achieve the same level of performance as structure-based models^43,44^, and can still require structural knowledge a priori, such as the location of the ligand binding pocket^43^.

In a parallel effort, Li and colleagues recently used a template-based modelling strategy to generate 3D structures for ligands with known binding affinities to target proteins in the PDBbind dataset^34^. This approach, described in greater detail in the original publication^34^ and the “Methods”, resulted in an augmented dataset of 69,816 additional complexes with modelled structures and experimental binding data, denoted as “BindingNet". Crucially, as the compounds included in BindingNet must bind a target that exists in PDBbind v2019, while also having sufficient structural similarity to the cognate ligand, this additional data increases ligand diversity and coverage. BindingNet also includes 51,131 activity cliffs (defined based on matched molecular pairs with at least a ten-fold potency difference) for 455 targets^34^. Following a similar approach, augmented data has also been generated by Gilson and colleagues as part of the BindingDB project^35^. Specifically, the BindingNet Docked Congeneric Series (DCS) dataset provides structures for protein–ligand complexes generated computationally using the Surflex docking program^45^. As with BindingNet, this process relies on an available crystal structure of a congeneric ligand to serve as a template for pose generation. In total, BindingDB-DCS contains 8822 protein–ligand complexes from 1322 congeneric series, further increasing ligand coverage.

**Fig. 3:**
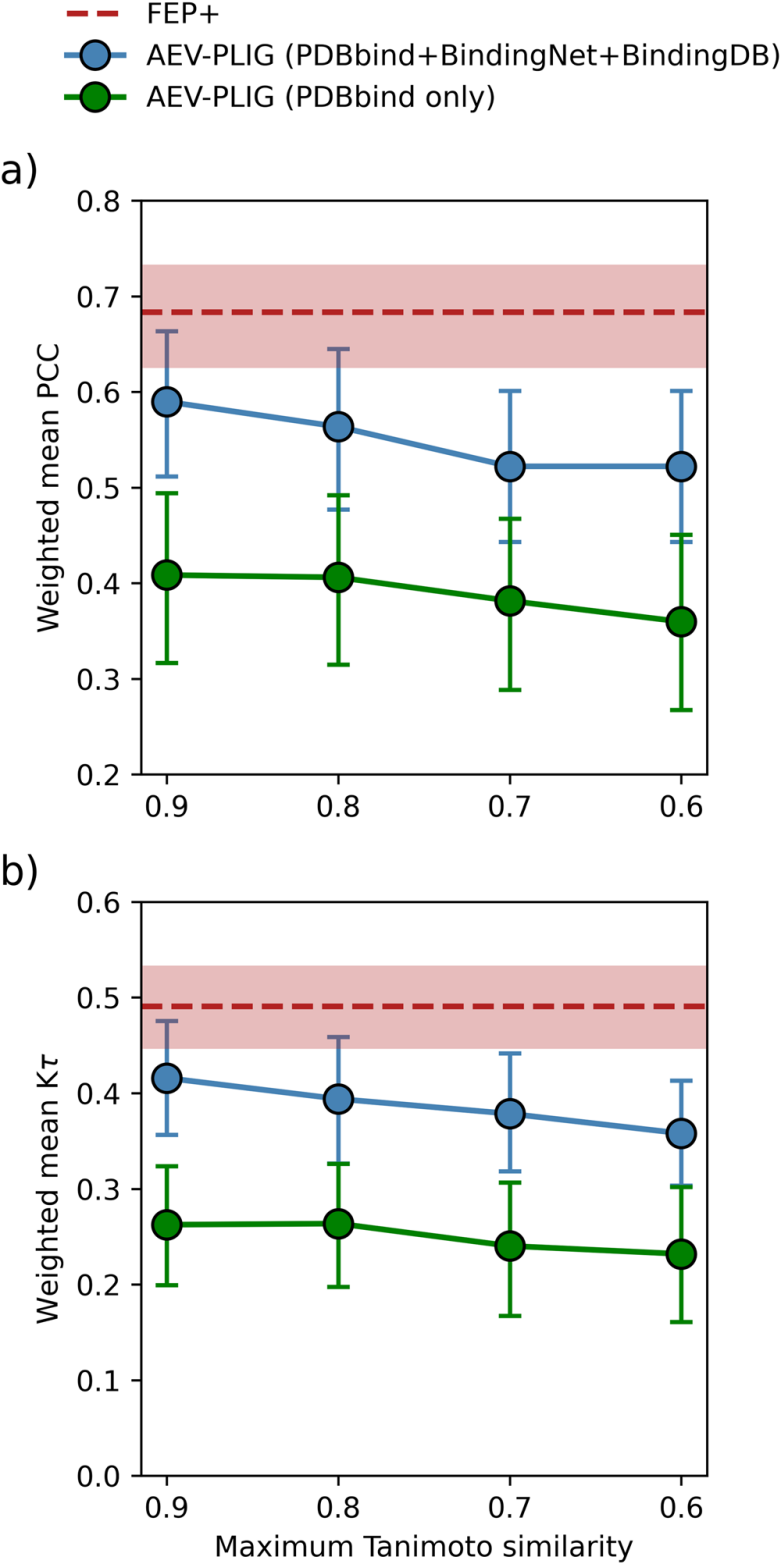
Performance of AEV-PLIG models on the FEP benchmark. Model performance in terms of **a** weighted mean PCC or **b** weighted mean K*τ*, as a function of maximum ligand Tanimoto similarity (*T*_s_) between the training and test sets. AEV-PLIG models were trained on PDBbind v2020 only (green) or PDBbind (2020) plus BindingNet and BindingDB-DCS. The performance of FEP+ on the FEP benchmark is included in red. Tanimoto similarity was computed using ECFP6 fingerprints and error bars represent 95% BCa bootstrap confidence intervals.

In light of these augmented datasets, we investigated the effect of augmenting the training of AEV-PLIG models using PDBbind v2020 together with BindingNet and BindingDB-DCS data, evaluating their performance on each benchmark described previously. For the CASF-2016, OOD Test and 0 Ligand Bias benchmarks, we found no significant improvement in performance when training with this additional data (Fig. S4). However, for the FEP benchmark, augmented data offered a substantial performance improvement for most systems individually and on average, with weighted mean PCC and K*τ* values increasing from 0.41 to 0.59 and from 0.26 to 0.42, respectively (Fig. 3). We established this improvement to be statistically significant (*P* < 0.0001, with a significance threshold of *P* < 0.05) by performing hypothesis tests on the differences in weighted mean PCC (and weighted mean K*τ*) values between the two models (see “Methods” for details). The performance of AEV-PLIG and FEP+ for each system in the FEP benchmark are also displayed in Fig. S2. The different effects of augmented data on model performance across these benchmarks can be examined by considering the nature of the data in the training and test sets. The FEP benchmark comprises many proteins each bound with a series of ligands, whereas CASF-2016 and OOD Test is composed of independent protein–ligand pairs, and 0 Ligand Bias features highly similar ligands bound to a variety of different proteins. The distribution of ligands and targets in the FEP benchmark is thus closely mimicked by the additional training data, where there is a high coverage of ligands per protein. In contrast, the OOD test and 0 Ligand Bias include, by design, proteins that are dissimilar to PDBbind and low coverage of ligands per protein, respectively.

Recognising that the presence of similar structures in the training set can lead to enhanced test performance, we sought to investigate the effects of ligand similarity on AEV-PLIG performance by removing structurally similar compounds from the training data. Specifically, we removed all complexes from the training set where the ligand Tanimoto similarity (*T*_s_) compared to each test set ligand exceeded 0.9, 0.8, 0.7 or 0.6, before training models on the remaining test-set dissimilar data. The performance metrics shown in Fig. 3 reveal that the AEV-PLIG models’ accuracy does indeed depend on the presence of similar ligands in the training data, although this dependence is modest at the levels of similarity investigated here. For example, removing ligands with *T*_s_ > 0.6 decreases performance by approximately 0.05 correlation units (PCC, K*τ*) compared to models trained on ligands with Tanimoto similarity up to 0.9. Nonetheless, even using a strict similarity threshold of 0.6, training AEV-PLIG with BindingNet and BindingDB-DCS still yields improvements in average performance for the FEP benchmark across all metrics relative to PDBbind v2020 (Fig. 3). To assess the impact of the quantity of augmented data on model performance, we also trained AEV-PLIG models using PDBBind v2020 supplemented with progressively larger fractions of data randomly sampled from the BindingNet and BindingDB-DCS datasets. Performance on the FEP benchmark increased with dataset size and, interestingly, did not saturate over the tested range, suggesting that incorporating additional augmented data could lead to future improvements (Fig. S5).

**Fig. 4:**
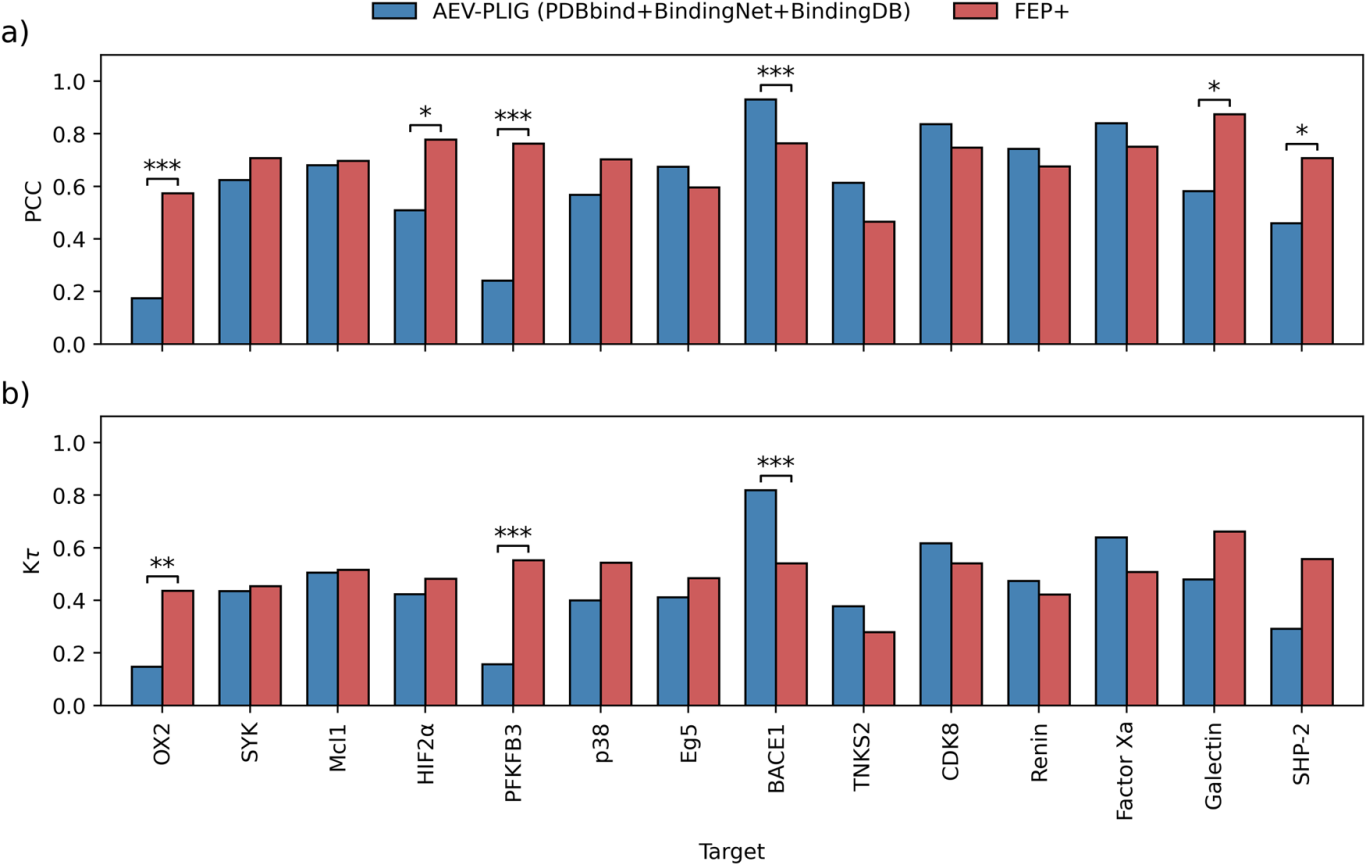
Performance comparison of FEP+ with AEV-PLIG models for targets in the FEP benchmark with 25 or more ligands. Comparison made in terms of **a** PCC and **b** K*τ*. AEV-PLIG models were trained on PDBbind v2020, BindingNet, and BindingDB-DCS (*T*_s_ ≤ 0.9). Error bars have been omitted for clarity. Asterisks indicate where two methods give a statistically significant performance difference (^*^P < 0.05, ^**^P < 0.01, ^***^P < 0.001), calculated as defined in the “Methods”.

Encouraged by these results, we sought to compare AEV-PLIG predictions for the FEP benchmark with those obtained using the FEP+ workflow. Overall, we found that the correlation and ranking ability of AEV-PLIG trained using PDBbind v2020, BindingNet and BindingDB (using *T*_s_ ≤ 0.9) was lower than FEP+ on average, providing a statistically significant difference in terms of mean weighted PCC (*P* = 0.012) and K*τ* (*P* = 0.014) across all series. To compare performance on individual targets, we further investigated the predictions for congeneric series comprising 25 or more ligands (14 targets in total, Fig. 4). To avoid the same target appearing twice in this set, the BACE1 series from the FEP+ R-group set was also excluded. We considered only these 14 targets with large numbers of ligands to ensure sufficient data for robust analysis. The outcomes for all systems in the FEP benchmark are provided in the Supporting Information (Fig. S2 and Table S3) and reflect the same conclusions. Across these 14 targets, prediction correlation and ranking metrics obtained using AEV-PLIG and FEP+ were comparable in 8/14 targets based on PCC; and for 11/14 targets for K*τ* values, respectively (Fig. 4). FEP+ exhibited superior correlation and ranking performance compared to AEV-PLIG for five (OX2, HIF2*α*, PFKFB3, Galectin, SHP-2) and two (OX2, PFKFB3) targets, respectively, whereas AEV-PLIG outperformed FEP+ for one series (BACE1) in terms of PCC and K*τ*. Significance was determined as described in the “Methods”. Scatter plots depicting both AEV-PLIG and FEP+ predictions for each of these 14 targets are provided in Fig. S6.

Given that FEP+ is able to sample multiple protein and ligand conformations via MD simulations, greater performance would be expected over ML models that use just a single “snapshot” to calculate binding affinity. However, ML offers significant advantages in terms of speed, convenience and flexibility. In particular, we highlight that on average, once trained, AEV-PLIG can generate a binding affinity prediction from a 3D structure in 33 ms with minimal computational requirements, whereas performing four ligand perturbations using FEP+ requires 24 h and eight commodity GPUs (Nvidia GTX-780)^9^, making AEV-PLIG at least  ~400,000 times faster than FEP+. In fact, even to train an entire AEV-PLIG model from scratch using PDBbind v2020 with augmented data ( ~95,000 data points) required just 28 h using a single GPU (Nvidia GTX-1080). Moreover, once trained AEV-PLIG offers a generalised approach with minimal system preprocessing, in contrast to FEP+, which depends on robust preparation and protocols that often need to be manually tailored to a given system. These factors highlight that ML-based scoring methods, exemplified by AEV-PLIG, can serve as complementary tools to MD-based methods, offering competitive performance in many cases, and at a fraction of the computational cost. While FEP can achieve superior accuracy through extensive conformational sampling and rigorous free energy calculations, AEV-PLIG offers an advantage where rapid predictions and scalability are paramount, such as ranking thousands of compounds within pre-existing libraries or sampled de novo using generative models.

## Conclusions

In this work, we have investigated a number of strategies to enhance the applicability of machine learning (ML) scoring functions and provide more realistic assessments of their performance, with a particular focus on drug discovery applications. We have also introduced a novel binding affinity prediction method, AEV-PLIG, that combines atomic environment vectors with protein–ligand interaction graphs, employing an attentional GNN architecture to capture the complex interplay of interactions that determine binding affinity. AEV-PLIG was evaluated alongside RFScore, Pafnucy, OnionNet-2, PointVS, SIGN, and AEScore, using a variety of benchmarks designed to assess performance on OOD data (OOD Test) and pharmaceutically-relevant systems (FEP benchmark), or penalise memorisation (0 Ligand Bias), revealing significantly worse performance than on the widely used benchmark for scoring functions, CASF-2016. When comparing different ML models, AEV-PLIG performed well, but no single model achieved significantly better performance across all benchmarks, highlighting the need for a diverse set of test cases to evaluate new models, features and architectures.

Given the prevailing issue of data scarcity in biochemical domains, we explored the use of augmented data by training models on experimental binding data but 3D protein–ligand structures modelled using template-based alignment or docking. This data augmentation strategy proved to be an effective means of significantly improving AEV-PLIG’s ranking ability for systems encountered in drug discovery, where compounds are designed in series by introducing small structural modifications intended to increase their binding affinity towards a given target. Our analysis also suggests that additional augmented data could provide future performance gains; given the recent introduction of accurate (protein–ligand) structure prediction models such as AlphaFold 3^46^, Umol^47^ and NeuralPlexer^48^, such data could soon be readily available.

In light of the results presented here, we envisage that this data could be pivotal in enabling ML-based tools to achieve the accuracy of rigorous simulation-based methods such as FEP for predicting binding free energies. Our work shows that ML models such as AEV-PLIG are closing the gap to “gold-standard” alchemical methods such as FEP+, achieving comparable performance for many congeneric series in the FEP benchmark when models are trained using augmented datasets. However, FEP methods continue to advance over time so whether the gap will continue to close remains to be seen. Furthermore, ML-based methods like AEV-PLIG benefit from many practical advantages, such as generating predictions that are five orders of magnitude faster than MD-based workflows do not require manual reparametrisation, and are thus amenable to high-throughput virtual screening. Together, these new methods offer powerful and complementary approaches for accelerating early-stage drug discovery.

## Methods

### Data

#### Training sets

##### PDBbind

To train and validate our baseline AEV-PLIG model, and compare it to other state-of-the-art models, we used the PDBbind v2020 dataset^22^. PDBbind v2020 is a curated database of 19,443 protein–ligand crystal structures with associated experimentally determined inhibition constants, *K*_*i*_, dissociation constants, *K*_*d*_, or IC_50_ measurements. The “Refined” subset of PDBbind v2020 contains 5316 complexes that satisfy more stringent criteria including a minimum crystal structure resolution of 2.5 Å and only *K*_*i*_ or *K*_*d*_ binding affinity measurements. We processed PDBbind with RDKit v2023^49^, removing 135 complexes with ligands that could not be parsed with RDKit; included uncommon elements (i.e. not H, B, C, N, O, F, P, S, Cl, Br, or I); or had unknown bond types.

##### BindingNet

To expand the training data for AEV-PLIG models, we incorporated augmented structural data from BindingNet. Li et al.^34^ generated this dataset by first identifying candidate compounds from ChEMBL with binding activity data for protein targets in PDBbind v2019. These compounds were selected based on their similarity to crystallized ligands for each target. A template-based alignment protocol based on the maximum common substructure (MCS) between candidate compounds and the crystal ligands was used. 3D conformations of the ligands in the binding pocket were generated and scored using molecular mechanics with Generalized Born and Surface Area solvation (MM-GB/SA)^50^. The top ligand poses were selected based on the MM-GB/SA binding energy and the root-mean-square deviation (RMSD) of the MCS. This process resulted in 69,816 additional structures, spanning 802 unique targets in PDBbind v2019. A detailed description of the procedure is given in Li et al.^34^. When processing this dataset for AEV-PLIG, we excluded 89 ligands containing uncommon elements (i.e. elements other than H, B, C, N, O, F, P, S, Cl, Br, or I).

##### BindingDB

We also included augmented data from the BindingDB Docked Congeneric Series (DCS) dataset, which contains a series of ligands that bind to the same protein with experimental binding affinity data and ligand poses that have been generated using molecular docking^35^. Similar to BindingNet, at least one experimental crystal structure of the target was required to serve as a template for docking. Congeneric series were identified automatically based on the Tanimoto similarity of ChemAxon fingerprints, while docking was performed using the Surflex-Dock program^45^. We processed the data by removing data points with ambiguous binding affinity measurements (e.g. measurements containing “>” and “<”) and ligands that could not be read using RDKit. BindingDB-DCS contains binding affinity *K*_*d*_, *K*_*i*_, and IC_50_ measurements. If a single datapoint includes multiple measurements, the median binding affinity was used, prioritising *K*_*d*_, then *K*_*i*_, and lastly IC_50_. After processing, BindingDB-DCS contained a total of 8822 protein–ligand complexes generated from 1311 unique PDB IDs.

#### Benchmarks

To ensure a comprehensive comparison of our models with other state-of-the-art scoring functions, we used five different benchmarks that are described in detail in this section, namely: “Comparative Assessment of Scoring Functions (CASF-2016)”^23,24^, OOD Test, 0 Ligand Bias^41^, FEP benchmark^10^. Details about the training and test sets are provided in Table 2. For the CASF-2016 and 0 Ligand Bias benchmarks, the “General set” of PDBbind v2020 was used for training, with any systems present in both PDBbind v2020 and the test sets being removed to ensure there was no “hard overlap”. For the FEP benchmark, we also removed complexes from the training data set based on their ECFP6 fingerprint similarity to test set ligands, using an upper limit of 0.9 for Tanimoto similarity, *T*_*s*_, unless otherwise stated. A subset of the General set of PDBbind v2020, which we call “Refined2020+”, was used to train models for the OOD test benchmark and is described in more detail below.

**Table 2:** Summary of the training and benchmark datasets used in this work

Dataset	Purpose	No. data points	Source
PDBbind v2020	Train	19,443	Ref. ^22^
Refined2020+	Train	18,402	This work
BindingNet	Train	69,816	Ref. ^34^
BindingDB-DCS	Train	8822	Ref. ^35^
CASF-2016	Test	285	Refs. ^23,24^
OOD Test	Test	295	This work
0 Ligand Bias	Test	365	Ref. ^41^
FEP benchmark	Test	948	Ref. ^10^

##### CASF

Developed for the Comparative Assessment of Scoring Functions (CASF), the CASF-2016 benchmark represents a curated subset of 285 complexes from the Refined subset of the PDBbind dataset v2016^23,24^. Briefly, proteins in PDBbind v2016 were first clustered by sequence identity using a cutoff of 90%. Five representative complexes were then selected from each cluster, including the most-active and least-active ligands alongside three additional complexes^24^. A more complete description of the selection procedure can be found in the original publication^24^.

##### OOD Test

In order to estimate generalisability, we propose an intuitive and simple way to quantify the OOD performance of scoring functions with a novel training and test set split of PDBbind v2020. The test set, called “OOD Test”, was constructed by minimising the similarity of the complexes in the OOD Test to the rest of the Refined set of PDBbind v2020 in terms of ligand Tanimoto similarity, *T*_*s*_, sequence identity *s*, and protein pocket similarity.

We calculated sequence identity using MMseqs2^51^, and the Tanimoto similarity using ligand ECFP6 fingerprints generated with RDKit v2023^49^. We first clustered the Refined set of PDBbind v2020 using agglomerative clustering with single linkage and a distance threshold of 0.5, where the distance between two complexes, *c*_1_ and *c*_2_, is defined as follows:

1 $$d({c}_{1},{c}_{2})=1-\max (s({c}_{1},{c}_{2})/100,{T}_{s}({c}_{1},{c}_{2})).$$

In cases where there are multiple protein sequences in one complex then the maximum sequence identity of any two protein sequences was used. We then sampled these clusters into the OOD test; to ensure that ligands in the OOD test satisfy simple criteria for drug-likeness, we excluded clusters that included heavy ligands (molecular weight > 1000 Da) or any ligands with more than 20 rotatable bonds. To ensure there was no pocket-specific similarity between proteins, we removed any clusters in the OOD Test with pocket Pfam cluster overlap with the training data. The pocket Pfam clusters were defined by Zhu et al.^52^. Briefly, their clustering procedure includes assigning PDBbind complexes a Pfam entry utilising the protein family database^53^ and Pfam entries in the PDB^54^. For proteins with multiple Pfam entries, the Pfam entry that shared the most residues with the ligand-binding pocket was used, denoted as the “pocket Pfam”. To cluster complexes that lacked a pocket Pfam entry, the protein sequences were iteratively aligned with pocket Pfam sequences of other proteins with known entries.

OOD Test consists of 295 complexes that have *T*_*s*_ < 0.5 and sequence identity < 50% to any other ligand/protein in the PDBbind v2020 Refined set. Additionally, the complexes in the OOD Test have no pocket Pfam cluster overlap with complexes in the training data. To maximise the number of training data points, we added all complexes from the General set of PDBbind v2020 that also have *T*_*s*_ < 0.5, and  <50% sequence identity to any complex in the OOD Test, and do not belong to the same pocket Pfam cluster as complexes in OOD Test. In total, we added 13,385 complexes to the training data. The final subset of PDBBind v2020 consists of 18,838 data points, herein referred to as “Refined2020+” (Table 2). The OOD Test benchmark training and test splits are accessible via: https://github.com/isakvals/OOD-Test.

##### 0 Ligand Bias

To ensure scoring functions utilise both protein and protein–ligand interaction features for accurate predictions, Durant et al. constructed a test set of 365 complexes from the PDBbind v2020 dataset called “0 Ligand Bias”^41^. The test set was constructed by clustering ligands by their InChI-Key^55^, creating clusters of the same ligand bound to various targets, with different clusters representing distinct ligands. The set consists of 90 clusters where the mean binding affinity is between 6 pK and 7 pK units and has a standard deviation greater than 1 pK unit. This construction ensures that models that learn only ligand-based features cannot rank the binding affinity of the test complexes. For more details, readers are referred to the original publication^41^.

##### FEP Benchmark

To evaluate our models on data representative of drug discovery projects, we used the comprehensive FEP benchmark set published by Ross et al.^10^. This dataset includes congeneric series from a wide range of previously published academic and industrial FEP studies, covers a variety of targets (e.g. GPCRs and kinases) and ligand modifications frequently encountered in drug discovery projects. The target, data source, and a number of ligands for each series are shown in Table S2. For each system, the FEP+ workflow was applied to predict the relative Gibbs free energy of binding, ΔΔ*G*_pred_, between pairs of ligands. Details about the system preparations and FEP+ protocols can be found in the original publication^10^.

As binding affinities are measured experimentally as *K*_*i*_, *K*_*d*_, or IC_50_ values, they must be converted to experimental free energies Δ*G* to facilitate comparison with FEP+. Denoting any of *K*_*i*_, *K*_*d*_, or IC_50_ as *K*, and following the same approach used by Wang et al.^9^ and Schindler et al.^56^, Δ*G* is calculated as:

2 $$\Delta G=RT\ln (K)$$

In contrast, ML models are typically trained to predict the negative logarithm of (absolute) experimental binding affinity, *pK*, which is proportional to Δ*G*:

3 $${{\rm{pK}}}=-\frac{1}{\ln (10)}\frac{\Delta G}{RT}$$

where *R* = 1.987 × 10^−3^ kcal K^−1^ mol^−1^ is the universal gas constant and *T* = 297 K is the absolute temperature. To convert pK values to Δ*G* values for comparison with FEP, we used the following formula:

4 $$\Delta G=-\ln (10)RT{{\rm{pK}}}$$

### Models

#### Atomic environment vectors

An atomic environment vector (AEV) is a vector that describes the local chemical environment of a reference atom, introduced by Smith et al.^39^ which was built on the pioneering work of Behler and Parinello^31^. Single-atom AEVs were employed to predict DFT potentials of organic molecules with the Accurate NeurAl networK engINe for Molecular Energies method, ANAKIN-ME or “ANI” for short. AEVs consist of two parts: radial and angular symmetry functions, which describe two-atom and three-atom interactions, respectively. In this work, we incorporated the radial part of the AEV (radial AEV) into our graphs, which is calculated for each ligand heavy atom node in the ligand GNN to describe the protein atoms that surround it.

For a given protein–ligand complex, let ${{{\mathcal{I}}}}_{{{\rm{ligand}}}}$ be the index set of the ligand heavy atoms in the complex, and ${{{\mathcal{I}}}}_{{{\rm{protein}}},t}$ be the index set of atoms of type $t\in {{\mathcal{T}}}$ in the protein, where ${{\mathcal{T}}}$ is the set of protein atom types. Let us also denote the radial AEV of an atom $i\in {{{\mathcal{I}}}}_{{{\rm{ligand}}}}$ with ${G}_{i}^{{{\rm{Rad}}}}$. The radial atomic environment of a reference atom is described using Gaussian functions centred at various distances from the atom. Further, the decreasing effect of atomic interactions with distance is implemented with a continuous cutoff function:

5 $${f}_{c}({R}_{ij})=\left\{\begin{array}{ll}\frac{1}{2}\left(\cos \left(\frac{\pi {R}_{ij}}{{R}_{c}}\right)+1\right),\quad &\,{\mbox{for}}\,\,{R}_{ij}\le {R}_{c}\\ 0\quad \hfill &\,{\mbox{for}}\,\,{R}_{ij} \, > \, {R}_{c},\end{array}\right.$$

where *R*_*ij*_ is the distance between the interacting atoms *i* and *j*, and *R*_*c*_ is a predefined cutoff radius. The cutoff function decays from 1 to 0 as *R*_*ij*_ increases, and is set to zero when the distance is larger than *R*_*c*_ (which we define as 5.1 Å; see Table 3). The radial AEV ${G}_{i}^{{{\rm{Rad}}}}$ consists of entries ${g}_{i}^{t,m}$, which are indexed by both the protein atom type $t\in {{\mathcal{T}}}$, and a vector *m* = (*η*, *R*_*s*_). The range of values that parameters *η* and *R*_*s*_ can take are also predefined. Each entry of ${G}_{i}^{{{\rm{Rad}}}}$ is then computed in the following way:

6 $${g}_{i}^{t,m}={\sum}_{j\in {{{\mathcal{I}}}}_{{{\rm{protein}}},{{\rm{t}}}}}{e}^{-\eta {({R}_{ij}-{R}_{s})}^{2}}{f}_{c}({R}_{ij}).$$

**Table 3:** The set of parameters used to construct the radial AEVs

Parameter	Values
*R*_*c*_ [Å]	5.1
*R*_*s*_ [Å]	0.80, 1.07, 1.34, 1.61, 1.88, 2.14
	2.41, 2.68, 2.95, 3.22, 3.49,
	3.76, 4.03, 4.29, 4.56, 4.83
*η*	19.7

These are the same parameters used in ANI-2x^57^.

**Fig. 5:**
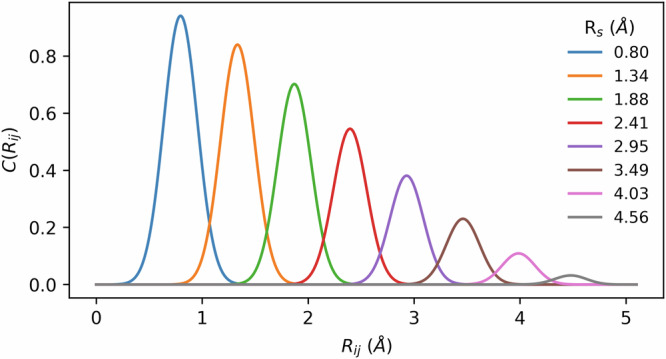
Radial AEV contributions of the atom $j\in {{{\mathcal{I}}}}_{{{\rm{protein,t}}}}$ for different values of *R*_*s*_, where *η* = 19.7 and *R*_*c*_ = 5.1 Å. The plot is inspired by a figure in ref. ^39^.

Here, *R*_*s*_ is the distance from the reference atom, and it defines the peak of the Gaussian function, whereas the width of the distribution is controlled by *η*. Let $N:= | {{\mathcal{T}}}|$ be the number of distinct atom types in the protein, *L* be the number of values *R*_*s*_ can take, and *K* be the number of values *η* can take. Then, the vector *m* from Eq. (6) can take *LK* different values, and hence the length ${G}_{i}^{{{\rm{Rad}}}}$ is *NLK*. The ANI papers^39,57^ utilise many narrow Gaussian functions by fixing a small *η* value, while letting *R*_*s*_ take different values between 0 and *R*_*c*_ (Fig. 5). This approach results in a fine resolution of the radial atomic environment, while restricting the size of the vector. Figure 5 shows the contributions to a radial AEV entry, which are defined as:

7 $$C({R}_{ij},\eta ,{R}_{s},{R}_{c})={e}^{-\eta {({R}_{ij}-{R}_{s})}^{2}}{f}_{c}({R}_{ij}).$$

#### Graph construction

The featurisation of the protein–ligand complex in this work was inspired by protein–ligand Interaction Graphs^37^ (PLIGs), developed by Moesser et al.^37^. The graphs in PLIGs have the topology of the ligand, with nodes representing ligand-heavy atoms, and edges representing ligand covalent bonds. For each ligand-heavy atom, their non-covalent interactions with the protein are encoded into the node feature vector by counting protein atoms closer than 6 Å, with Extended Connectivity Interaction Features (ECIF)^38^ atom typing being used to distinguish the protein atoms. A drawback of this representation is that it does not distinguish between a protein atom at different distances from the ligand atom within this cutoff. In this work, we improve upon PLIGs by utilising radial AEVs instead of atom counts, providing a richer description of protein–ligand interactions, whilst still ensuring the graph representation remains invariant to translation, rotation or mirror operations applied to the input structure.

For a protein–ligand complex, an AEV-Protein Ligand Interaction Graph (AEV-PLIG) is constructed by calculating a radial AEV for each of the ligand’s heavy atoms, using contributions from nearby protein atoms only. For the calculation of radial AEVs, we utilise the ECIF^38^ atom type definitions, which consist of 22 distinct protein atom types, i.e. $N:= | {{\mathcal{T}}}| =22$. The protein atom types from ECIFs are distinguished by the chemical element and five other properties: explicit valence, the number of attached heavy atoms, the number of attached hydrogen atoms, aromaticity, and ring membership. The radial AEVs are calculated with parameters from ANI-2x^57^ (Table 3), resulting in vectors with dimensions *NLK* = 22 × 16 × 1 = 352. The ligand-heavy atoms become nodes in the graph, and edges are added to represent covalent bonds. Both the radial AEVs and ligand atom descriptors are added to the node feature vectors. The ligand atom descriptors include the same five properties that define the ECIF atom types, as well as one-hot encodings of the chemical elements B, C, N, O, F, P, S, Cl, Br, and I. Finally, one-hot encodings of the bond orders (i.e. single, aromatic, double, and triple bonds) are assigned to the ligand’s edge features.

#### Neural network architecture

The neural network architecture we use to predict binding affinity is a GNN with five graph attention network (GAT) v2 layers^33^, each with three heads, followed by global pooling and a multi-layer perceptron (MLP) as depicted in Fig. 1. GATv2 is an attentional GNN which is a modified version of GATs^40^ that makes them strictly more expressive. Specifically, the updated feature vector on a node, *u*, is the weighted average of the linearly transformed features of the neighbouring nodes, after the application of a nonlinearity, *σ*:

8 $${h}_{u}=\sigma \left({\sum}_{v\in {{{\mathcal{N}}}}_{u}\cup \{u\}}{a}_{uv}{{{\bf{W}}}}_{t}{x}_{v}\right),$$

Here, *a*_*uv*_ is the attention coefficient with the following definition:

$${a}_{uv}=\frac{\exp \left({{{\bf{b}}}}^{\top }{{\rm{LeakyReLU}}}\left({{{\bf{W}}}}_{s}{x}_{u}+{{{\bf{W}}}}_{t}{x}_{v}+{{{\bf{W}}}}_{e}{e}_{uv}\right)\right)}{{\sum }_{k\in {{{\mathcal{N}}}}_{u}\cup \{u\}}\exp \left({{{\bf{b}}}}^{\top }{{\rm{LeakyReLU}}}\left.\left({{{\bf{W}}}}_{s}{x}_{u}+{{{\bf{W}}}}_{t}{x}_{k}+{{{\bf{W}}}}_{e}{e}_{u,k}\right]\right)\right)},$$

where **W**_*s*_, **W**_*t*_, **W**_*e*_, and **b** are learnable weight matrices shared over the layer. In attentional layers, *a*_*uv*_ can be interpreted as the importance of node *v* to the representation of node *u*, and is feature-dependent so it allows for learnable importance. Veličković et al.^40^ also extend Eq. (8) to implement *multi-headed attention*, which was originally introduced by Vaswani et al.^58^. Multi-headed attention concatenates multiple attention mechanisms from Eq. (8), with each attention mechanism learning different weight matrices.

As the node feature vectors are propagated through the GNN architecture, we encode the updated features as 256-dimensional vectors for each of the three heads. We summarise the final graph representation with global max and mean pooling, followed by a 4-layer MLP with dimensions 1590, 1024, 512, 256, and finishing with a 1-dimensional output.

#### Training and evaluation

All our AEV-PLIG models were trained independently ten times with different random seeds, and the mean predictions of the ten models are reported. Each model trained on PDBbind v2020 or Refined2020+ was trained for 300 epochs, whereas models trained with the addition of BindingNet and BindingBD-DCS were trained for 200 epochs. The model parameters for the epoch with the highest PCC on a validation set were saved. A list of the hyperparameters is reported in Table S1. All code for data processing and model training can be accessed here: https://github.com/oxpig/AEV-PLIG.

We investigated how a variety of state-of-the-art machine-learning scoring functions compare to AEV-PLIGs in terms of predicting binding affinity on five different benchmarks. Durant et al.^41^ provide a platform called ToolBoxSF for executing scoring functions, which we used to train five models: one random forest, two CNNs and two GNNs, namely RF-Score^15^, Pafnucy^59^, OnionNet-2^60^, PointVS, and SIGN^61^. We also compared our method with AEScore^32^.

#### Computational requirements and performance

The training process for AEV-PLIG (including training ten independent models) using PDBbind v2020 and BindingNet required approximately 28 h on a single NVIDIA GeForce GTX 1080 Ti GPU. Data processing and binding affinity prediction for 100 complexes took 3.25 s on a Mac M1 CPU, averaging 33 ms per complex.

#### Performance metrics

To evaluate the performance of the binding affinity prediction methods, we primarily used the Pearson correlation coefficient (PCC); and Kendall’s *τ* correlation coefficient (K*τ*), which captures the rank-order correlation between predicted and experimental values. While the prediction root mean square error (RMSE) is reported on occasion (Table S3), it is less informative for prospective drug discovery applications, where decisions are typically informed by the relative ordering of compounds; indeed, for test series with a low range of affinity values, low RMSE (≤ 1 kcal/mol) can be achieved with predictions that are only weakly correlated to the experimental values (e.g. see misc/hiv_prot_ekegren, Table S3).

##### Pearson correlation coefficient

PCC measures the linear relationship between two variables, and is defined as:

9 $${{\rm{PCC}}}=\frac{\mathop{\sum }_{i = 1}^{n}({x}_{i}-\bar{x})({y}_{i}-\bar{y})}{\sqrt{\mathop{\sum }_{i = 1}^{n}{({x}_{i}-\bar{x})}^{2}\mathop{\sum }_{i = 1}^{n}{({y}_{i}-\bar{y})}^{2}}}$$

where *x*_*i*_ and *y*_*i*_ are the experimental and predicted affinities, respectively, $\bar{x}$ and $\bar{y}$ are their mean values, and *n* is the number of data points. PCC ranges from −1 to 1, with 1 indicating perfect positive correlation, −1 indicating perfect negative correlation, and 0 indicating no linear correlation.

##### Kendall’s *τ* correlation coefficient

K*τ* assesses the ordinal association between two variables. It is calculated as:

10 $$\, {{\mbox{K}}}\,\tau =\frac{{n}_{c}-{n}_{d}}{\frac{1}{2}n(n-1)}$$

where *n*_*c*_ is the number of concordant pairs and *n*_*d*_ is the number of discordant pairs among the *n* pairs of predicted and actual binding affinities (i.e. sample size). A concordant pair occurs when the order of both elements in the pair is the same in both sets, while a discordant pair occurs when the order differs. K*τ* also ranges from −1 to 1, with 1 indicating perfect ranking agreement, −1 indicating perfect disagreement, and 0 indicating no association.

##### Statistical tests

To establish whether predictions generated using method *M*_1_ have significantly better correlation or ranking metrics than method *M*_2_, we use a bootstrapping procedure, defined as follows. Given the metric of interest (e.g. PCC or K*τ*) as *ρ*, we define the null hypothesis ${H}_{0}:{\rho }_{{M}_{1}}\le {\rho }_{{M}_{2}}$. For each of 10,000 boostrapping samples *b* ∈ [1, …, 10,000] we then calculate the difference in *ρ* between methods *M*_1_ and *M*_2_ for the same sample:

$$\Delta {\rho }_{b}={\rho }_{b,{M}_{1}}-{\rho }_{b,{M}_{2}}.$$

We used this test to compare AEV-PLIG trained with and without augmented data, and to compare AEV-PLIG to FEP+ in overall ranking ability and for each of the congeneric series in the FEP benchmark containing 25 or more ligands. *P*-values are reported as the fraction of bootstrap samples for which Δ*ρ*_*b*_ is negative, and we consider a *P*-value < 0.05 to indicate statistical significance.

## Supplementary information

Supplemental Information

## Data Availability

The OOD Test benchmark training and test splits are available in the OOD Test repository: https://github.com/isakvals/OOD-Test. All other training and benchmark data is publicly available and can be accessed via the respective publications.

## Code availability

The code used to process data and train AEV-PLIG models can be accessed in the AEV-PLIG repository licensed under the 3-Clause BSD License: https://github.com/oxpig/AEV-PLIG and forked at https://github.com/bigginlab/AEV-PLIG. The code used for data analysis and figure preparation is available in the repository for this manuscript: https://github.com/matthewtwarren/aev-plig-paper and also forked at https://github.com/bigginlab/aev-plig-paper.
