# Supplementary material for: Narrowing the gap between machine learning scoring functions and free energy perturbation using augmented data
Source: Commun Chem. 2025 Feb 8;8:41. doi: 10.1038/s42004-025-01428-y (PMC11807228; doi:10.1038/s42004-025-01428-y)
Supplement: Supplementary file 1 — Supplemental Information [file 42004_2025_1428_MOESM1_ESM.pdf]

Supporting Information: Narrowing the gap between machine learning scoring functions and free energy perturbation using augmented data

Ísak Valsson,[†,§] Matthew T. Warren,[‡,§] Charlotte M. Deane,[†] Aniket Magarkar,[*,¶] Garrett M. Morris,[*,†] and Philip C. Biggin[*,‡]

†*Department of Statistics, University of Oxford, 24-29 St Giles', Oxford, OX1 3LB, UK*

‡*Department of Biochemistry, University of Oxford, South Parks Road, Oxford, OX1 3QU, UK*

¶*Boehringer Ingelheim Pharma GmbH & Co. KG, Birkendorfer Str. 65, 88397 Biberach an de Riß, Germany*

§*These authors contributed equally to this work*

E-mail: aniket.magarkar@boehringer-ingelheim.com; garrett.morris@stats.ox.ac.uk; philip.biggin@bioch.ox.ac.uk

Supporting tables and figures

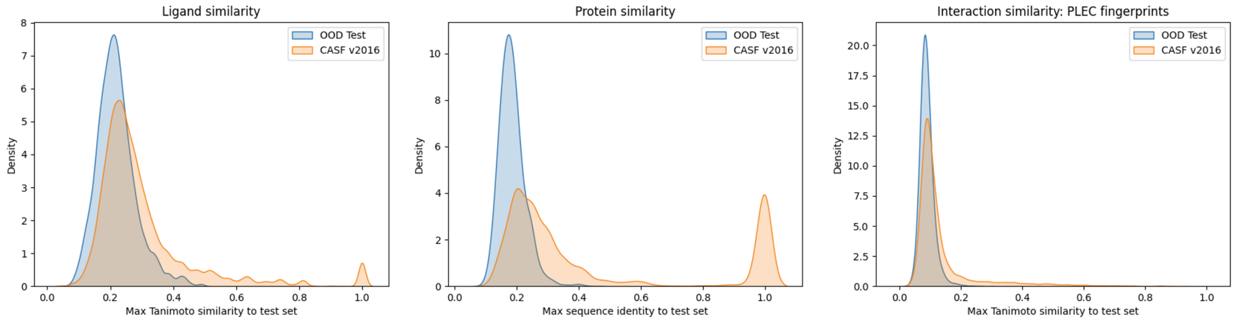

Figure S1: Maximum similarity of training complexes to test complexes in CASF v2016 (orange) and OOD Test (blue), in terms of ligand Tanimoto similarity (left), protein sequence similarity (middle), and protein-ligand interaction similarity (right). We calculated sequence identity with MMseqs2,[1] and ligand Tanimoto similarity using ECFP6 fingerprints generated with RDKit v2023.[2] We use Tanimoto similarity of PLEC[3] fingerprints to estimate protein-ligand interaction similarity.

Table S1: Hyperparameter values for AEV-PLIG trained on PDBbind v2020 only (left), and on PDBbind v2020 with augmented data from BindingNet and BindingDB-DCS (right).

**Parameter**	**Value**
Learning rate	$1.03 * 10^{-4}$, $1.23 * 10^{-4}$
Activation function	LeakyReLU, LeakyReLU
Batch size	32, 128

Table S2: Summary of FEP benchmark datasets, proteins and congeneric series. Adapted from Ref.[4]

**Data set name**	**Protein targets**	**N**
FEP+ R-group set	BACE1, CDK2, JNK1, Mcl1, p38, PTP1B, thrombin, TYK2	199
FEP+ charge-change	CDK2, DLK, EGFR, EPHX2, IRAK4, ITK, JAK1, JNK1 PTP1B, TYK2	53
OPLS stress set	BACE1, CHK1, Factor Xa	114
OPLS drug discovery	A, B, C, D, E	93
Water displacement	BRD4(1), CHK1, Hsp90, scytalone dehydratase, TAF1(2) thrombin, urokinase	76
FEP+ Fragments	T4 lysozyme, LigA, Mcl1, MUP-1, JAK-2, hsp90, p38	79
FEP+ macrocycles	BACE1, CHK1, CK2, MHT1, HSP90	34
FEP+ scaffold-hopping	BACE1, β-tryptase, CHK1, ERα, Factor Xa	17
Merck sets	CDK8, cMet, Eg5, HIF-2α, PFKFB3, SHP-2, SYK, TNKS2	264
GPCRs	A2A, OX2, P2Y1	98
Bayer macrocycles	Ftase, BRD4	8
Janssen BACE1	BACE1	74
MCS docking	HNE, Renin	49
Miscellaneous	CDK8, Galectin, BTK, HIV1 protease, FAAH	79
**Total**		**1237**

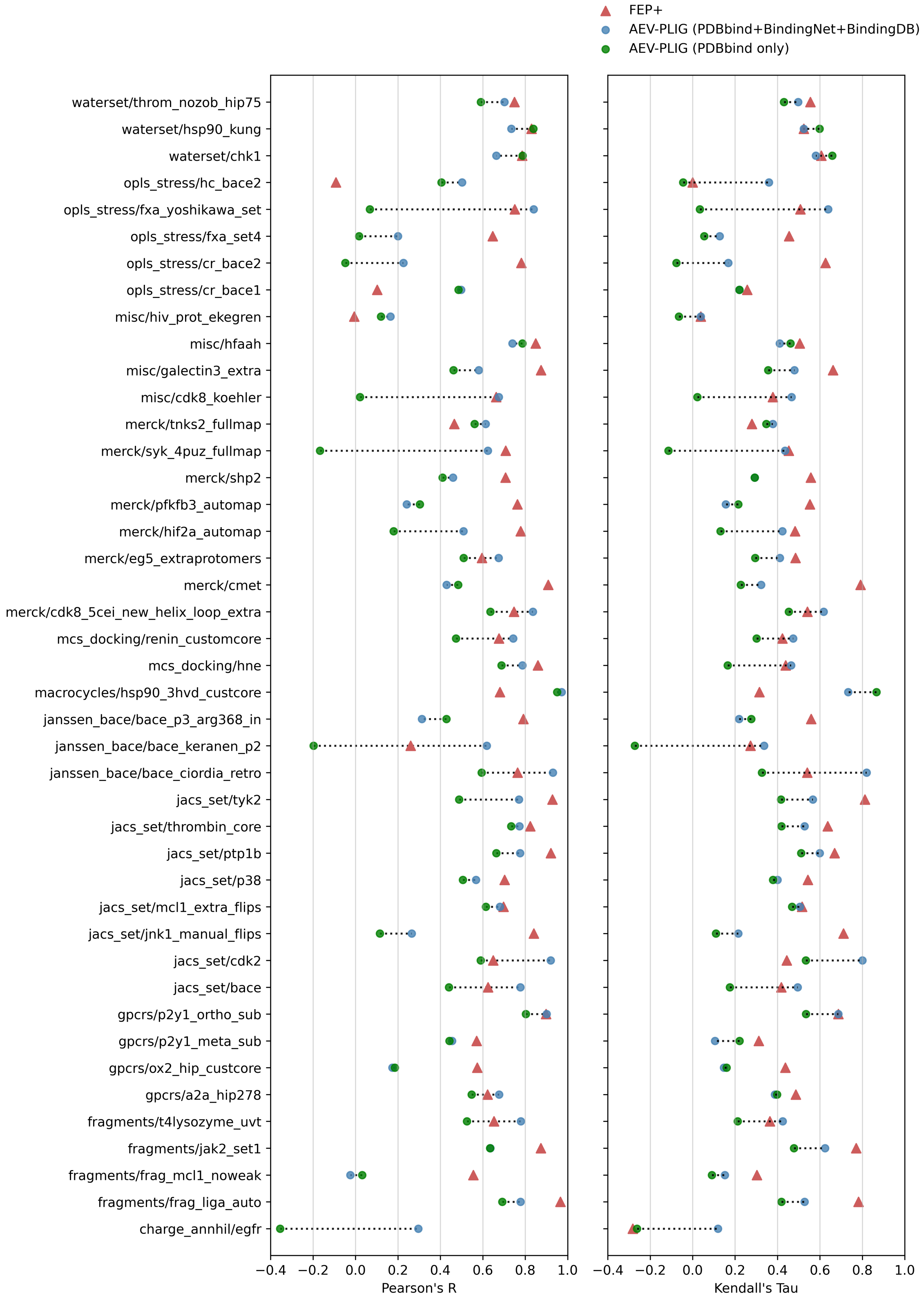

Figure S2: Performance of FEP+ and AEV-PLIG models on FEP benchmark dataset in terms of Pearson's correlation coefficient (left) and Kendall Tau (right).

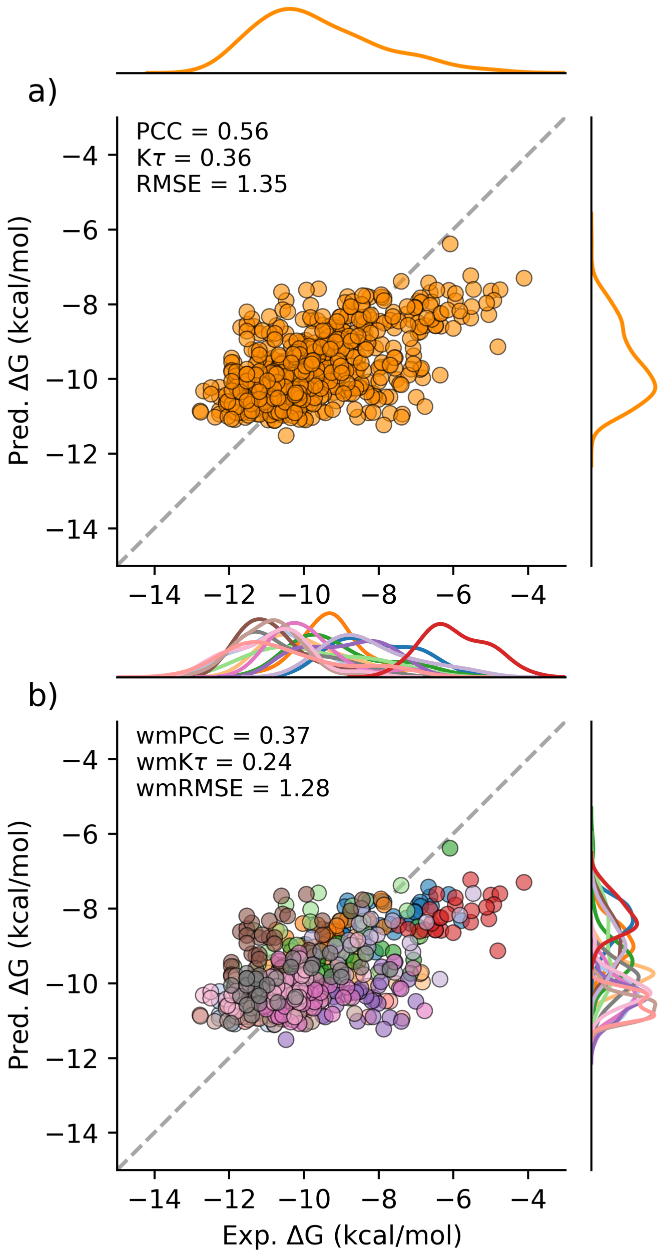

Figure S3: Scatter plots showing predicted vs. experimental binding free energies for FEP benchmark series containing 25 or more ligands. Predictions were obtained using AEV-PLIG models trained using PDBbind v2020 + BindingNet + BindingDB-DCS (using Tanimoto similarity ≤ 0.9). In panel a), scatter points, distributions, and performance metrics are shown for all series combined, whereas b) depicts each series individually and mean performance metrics weighted by the number of ligands in each series (i.e., wmPCC) are shown. RMSE is given in kcal/mol.

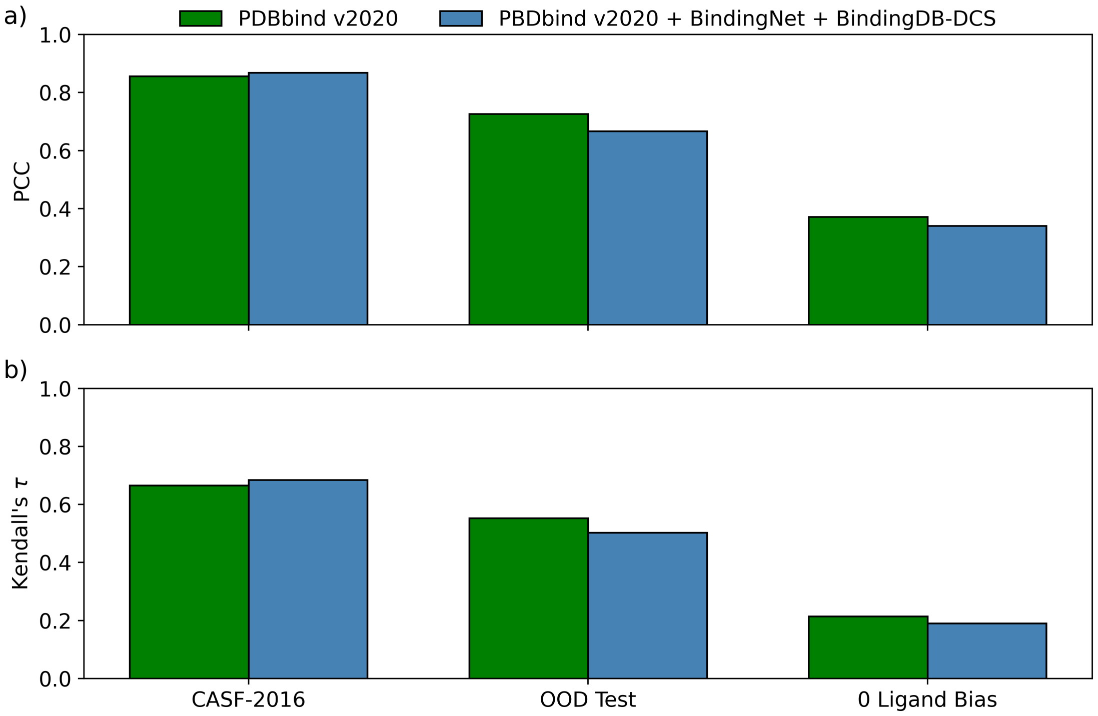

Figure S4: The performance of AEV-PLIG models trained using PDBbind v2020 or PBDbind v2020 + BindingNet + BindingDB-DCS, evaluated on different benchmarks. To incorporate the augmented data (BindingNet + Binding-DCS) into the benchmarks we did some processing to limit dataset leakage. For CASF-2016, we removed any datapoints labeled with PDB IDs in the CASF-2016 test set. For OOD Test, we only included augmented datapoints with PDB IDs in Refined2020+ and ensured that all added molecules had < 0.5 Tanimoto similarity to test set molecules. Finally, for 0 Ligand Bias we removed any datapoints labeled with PDB IDs from the test set, and removed complexes with identical molecules to any molecule in the test set. Results are shown in terms of (a) Pearson correlation coefficient (PCC), and (b) Kendall's τ.

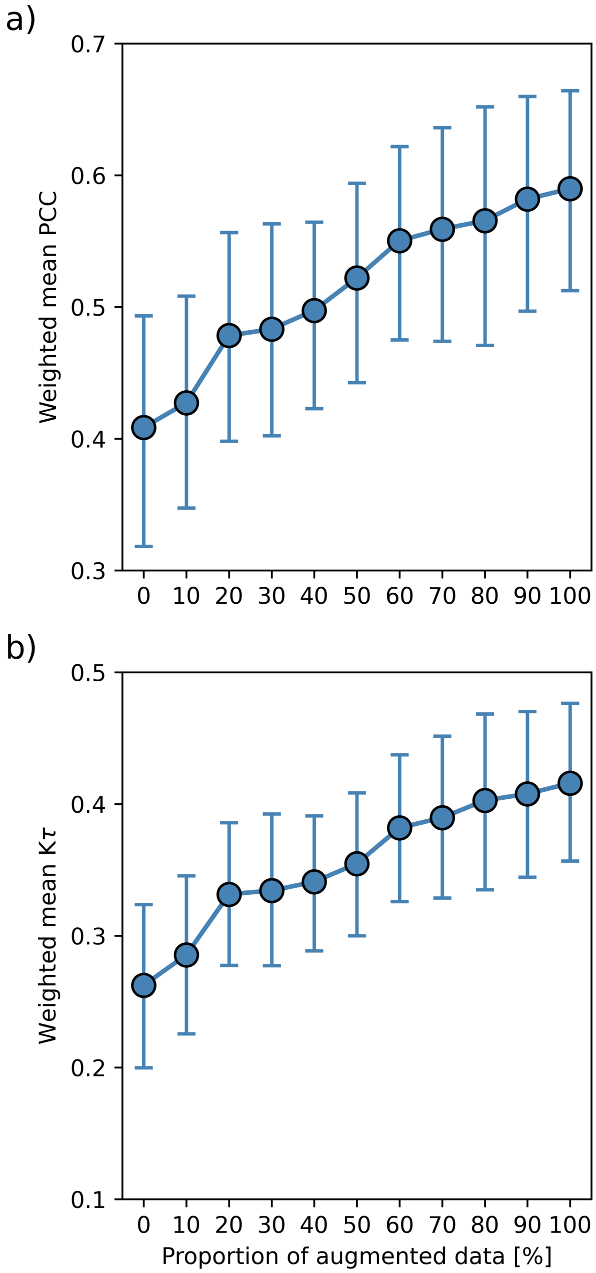

Figure S5: The performance of AEV-PLIG models trained using PDBbind v2020 with increasing fractions of augmented data. Models were evaluated on FEP benchmark using weighed mean PCC (a) and Kτ (b) metrics. The indicated fraction of augmented data was sampled by randomly selecting congeneric series from from BindingNet and BindingDB-DCS (Tanimoto similarity ≤ 0.9). Error bars represent 95% BCa bootstrap confidence intervals.

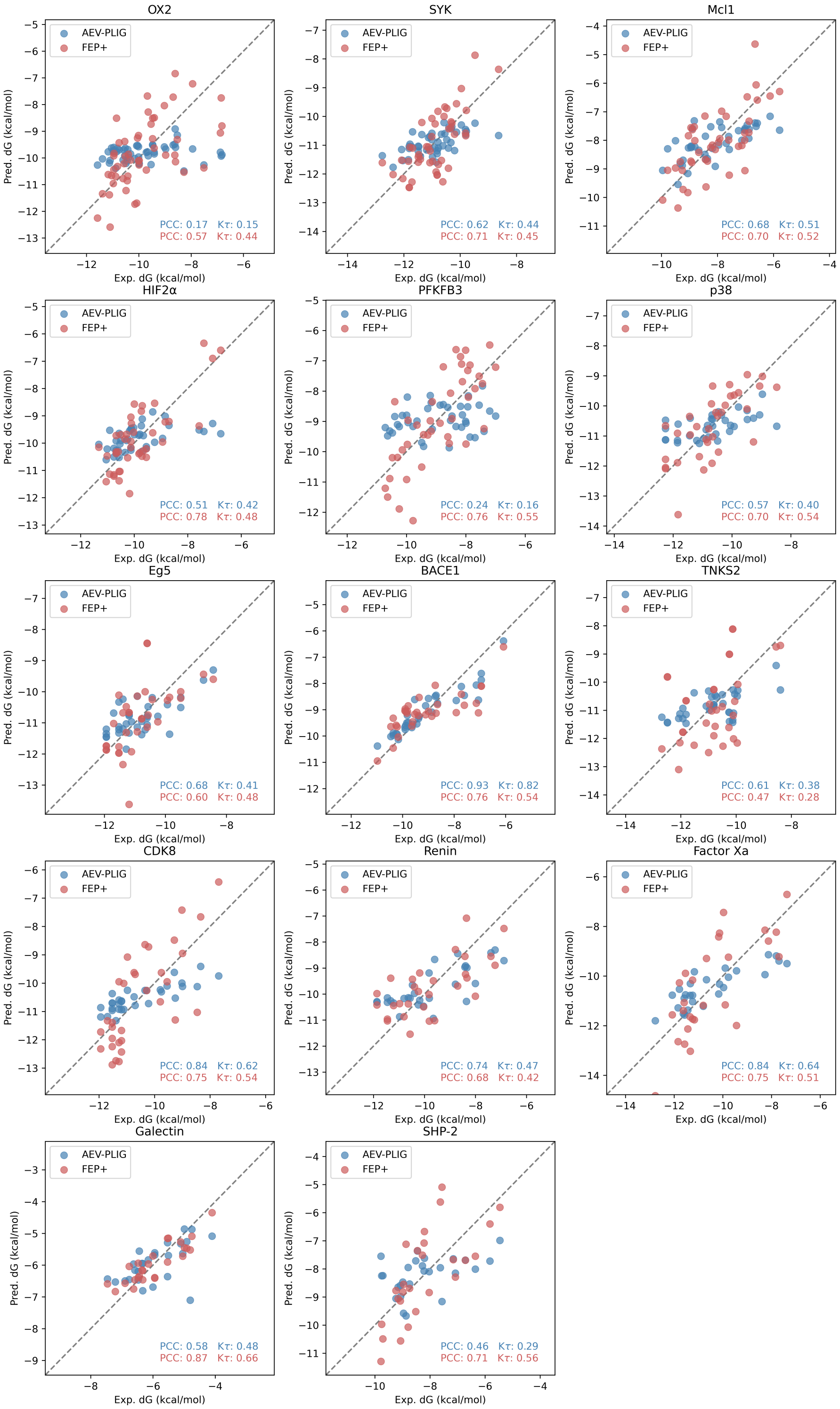

Figure S6: Scatter plots showing predicted vs. experimental binding free energies for FEP benchmark series containing 25 or more ligands. Predictions were obtained using AEV-PLIG models (blue) trained using PDBbind v2020 + BindingNet + BindingDB-DCS (using Tanimoto similarity ≤ 0.9) or FEP+ (red). AEV-PLIG results have been shifted so that the experimental mean is equal to the predicted mean. FEP+ results were obtained from Ref.[4]

Table S3: Performance metrics for the FEP benchmark congeneric series with ten or more ligands. Metrics for FEP+ were derived from data obtained from Ref.[4] AEV-PLIG models were trained using PDBbind (v2020) + BindingNet + BindingDB-DCS. $RMSE_{\Delta\Delta G}$ metrics were computed edgewise for edges obtained from Ref.[4] and for the same pairs across FEP+ and AEV-PLIG. [a,b]Incomplete data (edges/structures) and naming inconsistencies in Ref.[4] meant that in certain cases $RMSE_{\Delta\Delta G}$ could not be calculated[a] or does not include all edges[b]. RMSE is given in units of kcal/mol.

		FEP+				AEV-PLIG			
**System**	**N**	**PCC**	**K_τ**	**$RMSE_{\Delta G}$**	**$RMSE_{\Delta\Delta G}$**	**PCC**	**K_τ**	**$RMSE_{\Delta G}$**	**$RMSE_{\Delta\Delta G}$**
gpcrs/ox2_hip_custcore	51	0.57	0.44	1.13	1.43	0.17	0.15	1.23	1.67
merck/syk_4puz_fullmap[b]	46	0.71	0.45	0.75	0.84	0.62	0.44	1.25	0.86
jacs_set/mcl1_extra_flips	42	0.70	0.52	0.86	1.50	0.68	0.51	0.81	1.01
merck/hif2a_automap	41	0.78	0.48	0.77	1.23	0.51	0.42	0.95	1.21
merck/pfkfb3_automap[b]	40	0.76	0.55	0.99	2.33	0.24	0.16	1.13	1.29
jacs_set/bace	36	0.62	0.42	0.75	1.04	0.78	0.50	0.84	0.62
jacs_set/p38	34	0.70	0.54	0.81	0.89	0.57	0.40	0.88	1.08
merck/eg5_extraprotomers	34	0.60	0.48	0.88	1.30	0.68	0.41	1.83	0.90
janssen_bace/bace_ciordia_retro	33	0.76	0.54	0.81	1.10	0.93	0.82	0.54	0.66
merck/tnks2_fullmap	33	0.47	0.28	1.23	1.59	0.61	0.38	1.17	1.00
merck/cdk8_5cei_new_helix_loop_extra[b]	32	0.75	0.54	1.13	1.77	0.84	0.62	1.27	0.80
mcs_docking/renin_customcore	29	0.68	0.42	1.04	1.80	0.74	0.47	1.26	1.42
opls_stress/fxa_yoshikawa_set	27	0.75	0.51	1.27	1.88	0.84	0.64	0.94	1.16
misc/galectin3_extra	26	0.87	0.66	0.42	0.64	0.58	0.48	1.61	0.87
merck/shp2[b]	26	0.71	0.56	1.11	1.35	0.46	0.29	1.15	1.22
misc/hfaah[a]	24	0.85	0.50	0.75		0.74	0.41	2.18	
merck/cmet	24	0.91	0.79	0.74	0.84	0.43	0.32	1.59	1.26
jacs_set/ptp1b	23	0.92	0.67	0.51	0.64	0.78	0.60	0.86	1.18
jacs_set/jnk1_manual_flips	21	0.84	0.71	0.57	1.31	0.26	0.21	0.82	0.85
janssen_bace/bace_p3_arg368_in	21	0.79	0.56	1.07	1.26	0.31	0.22	1.45	1.27
waterset/throm_nozob_hip75[b]	21	0.75	0.56	0.72	1.09	0.70	0.50	0.81	0.80
mcs_docking/hne	20	0.86	0.44	0.96	1.37	0.79	0.46	1.16	1.29
gpcrs/a2a_hip278[b]	20	0.62	0.49	1.12	2.07	0.68	0.39	2.65	0.89
gpcrs/p2y1_meta_sub	20	0.57	0.31	1.04	1.55	0.46	0.11	2.00	1.15
jacs_set/cdk2	16	0.65	0.44	0.90	1.16	0.92	0.80	0.94	0.77
jacs_set/tyk2	16	0.93	0.81	0.47	0.82	0.77	0.57	0.84	1.11
waterset/chk1	13	0.78	0.61	0.95	1.04	0.66	0.58	1.12	1.57
misc/hiv_prot_ekegren	13	-0.01	0.04	0.76	1.09	0.16	0.04	0.43	0.53
opls_stress/cr_bace2	12	0.78	0.63	0.86	1.15	0.23	0.17	8.97	1.84
fragments/t4lysozyme_uvt	12	0.65	0.36	0.61	0.88	0.78	0.42	0.48	0.56
fragments/frag_mcl1_noweak[b]	12	0.55	0.30	0.87	1.16	-0.02	0.15	1.10	1.10
janssen_bace/bace_keranen_p2[b]	12	0.26	0.27	0.40	0.53	0.62	0.34	0.90	0.28
gpcrs/p2y1_ortho_sub	12	0.90	0.69	0.86	1.21	0.90	0.69	1.83	0.77
opls_stress/cr_bace1	11	0.10	0.26	1.16	1.49	0.50	0.22	8.90	1.26
opls_stress/fxa_set4	11	0.65	0.45	0.96	1.20	0.20	0.13	1.50	1.46
waterset/hsp90_kung	11	0.83	0.52	1.14	1.51	0.73	0.52	1.22	1.14
jacs_set/thrombin_core	11	0.82	0.64	0.53	0.94	0.77	0.53	0.53	0.72
fragments/frag_liga_auto	11	0.97	0.78	0.62	0.92	0.78	0.53	1.35	1.41
fragments/jak2_set1[b]	11	0.87	0.77	0.55	0.72	0.64	0.62	0.95	1.27
opls_stress/hc_bace2	10	-0.09	0.00	1.04	1.42	0.50	0.36	0.80	0.66
macrocycles/hsp90_3hvd_custcore	10	0.68	0.31	1.34	1.47	0.97	0.73	0.75	0.59
charge_annhil/egfr	10	-0.45	-0.28	0.74	1.08	0.30	0.12	0.53	0.55
misc/cdk8_koehler	10	0.66	0.38	0.90	1.21	0.68	0.47	0.42	0.52

Supplementary References

(1) Steinegger, M.; Söding, J. MMseqs2 enables sensitive protein sequence searching for the analysis of massive data sets. *Nature Biotechnology* **2017**, *35*, 1026–1028.

(2) Landrum, G. RDKit: Open-source cheminformatics. v2023.03.01.

(3) Wójcikowski, M.; Kukiełka, M.; Stepniewska-Dziubinska, M. M.; Siedlecki, P. Development of a protein–ligand extended connectivity (PLEC) fingerprint and its application for binding affinity predictions. *Bioinformatics* **2018**, *35*, 1334–1341.

(4) Ross, G. A.; Lu, C.; Scarabelli, G.; Albanese, S. K.; Houang, E.; Abel, R.; Harder, E. D.; Wang, L. The maximal and current accuracy of rigorous protein-ligand binding free energy calculations. *Communications Chemistry* **2023**, *6*, 1–12, Number: 1 Publisher: Nature Publishing Group.
